# *Helicobacter pylori* Disrupts Host Cell Membranes, Initiating a Repair Response and Cell Proliferation

**DOI:** 10.3390/ijms130810176

**Published:** 2012-08-15

**Authors:** Li-Ling Lin, Hsuan-Cheng Huang, Satoshi Ogihara, Jin-Town Wang, Meng-Chuan Wu, Paul L. McNeil, Chiung-Nien Chen, Hsueh-Fen Juan

**Affiliations:** 1 Institute of Molecular and Cellular Biology and Department of Life Science, National Taiwan University, Taipei 106, Taiwan; E-Mail: f94b43019@ntu.edu.tw; 2 Institute of Biomedical Informatics, Center for Systems and Synthetic Biology, National Yang-Ming University, Taipei 112, Taiwan; E-Mail: hsuancheng@ym.edu.tw; 3 Biological Science, Graduate School of Science, Osaka University, Osaka 560-0043, Japan; E-Mail: ogihara@bio.sci.osaka-u.ac.jp; 4 Department of Microbiology, National Taiwan University College of Medicine, Taipei 100, Taiwan; E-Mails: wangjt@ntu.edu.tw (J.-T.W.); d94445008@ntu.edu.tw (M.-C.W.); 5 Department of Cellular Biology and Anatomy and Institute of Molecular Medicine and Genetics, Georgia Health Science University, 1120 15th St., Augusta, GA 30912, USA; 6 Angiogenesis Research Center, National Taiwan University, Taipei 106, Taiwan; 7 Department of Surgery, National Taiwan University Hospital, Taipei 100, Taiwan

**Keywords:** plasma membrane repair, *Helicobacter pylori*, cell proliferation, annexin A4

## Abstract

*Helicobacter pylori* (*H. pylori*), the human stomach pathogen, lives on the inner surface of the stomach and causes chronic gastritis, peptic ulcer, and gastric cancer. Plasma membrane repair response is a matter of life and death for human cells against physical and biological damage. We here test the hypothesis that *H. pylori* also causes plasma membrane disruption injury, and that not only a membrane repair response but also a cell proliferation response are thereby activated. Vacuolating cytotoxin A (VacA) and cytotoxin-associated gene A (CagA) have been considered to be major *H. pylori* virulence factors. Gastric cancer cells were infected with *H. pylori* wild type (vacA+/cagA+), single mutant (ΔvacA or ΔcagA) or double mutant (ΔvacA/ΔcagA) strains and plasma membrane disruption events and consequent activation of membrane repair components monitored. *H. pylori* disrupts the host cell plasma membrane, allowing localized dye and extracellular Ca^2+^ influx. Ca^2+^-triggered members of the annexin family, A1 and A4, translocate, in response to injury, to the plasma membrane, and cell surface expression of an exocytotic maker of repair, LAMP-2, increases. Additional forms of plasma membrane disruption, unrelated to *H. pylori* exposure, also promote host cell proliferation. We propose that *H. pylori* activation of a plasma membrane repair is pro-proliferative. This study might therefore provide new insight into potential mechanisms of *H. pylori*-induced gastric carcinogenesis.

## 1. Introduction

Plasma membrane disruption injury can be the result of a variety of environmental stressors, which may be mechanical-, toxin-, or pathogen-based. This potentially lethal cell injury rapidly evokes a survival response in the form of membrane repair, which is triggered by the entry of extracellular Ca^2+^ through the disrupted membrane [1–3]. The process of repair involves the homotypic fusion of cytoplasmic membranes in addition to exocytotic fusion; the net result is the formation of a reparative patch across the disruption site of the plasma membrane. This dynamic process is mediated by a number of proteins, with annexins playing an important role [2,4–8].

*Helicobacter pylori* is a gram-negative microaerophilic bacterium that colonizes the gastric epithelium [9]. Individuals infected with *H. pylori* are at an increased risk of developing gastritis and peptic ulcers. Moreover, *H. pylori* is the first bacterium to be recognized as a causative agent of gastric cancer [10]. Two major pathological proteins of *H. pylori* mediate the infection: vacuolating cytotoxin A (VacA) and cytotoxin-associated gene A (CagA). VacA is secreted by *H. pylori* and inserts itself into the plasma membrane of host gastric epithelial cells to induce cellular vacuolation and associated damage [11]. CagA is delivered from the attached *H. pylori* directly into the cytosol of the host gastric epithelial cells, where it triggers signal transduction events (e.g., proliferation and inflammation), leading to gastric disease [11,12].

Certain bacteria, such as *Salmonella enterica* serovar Typhimurium [3], *Yersinia pseudotuberculosis* [3], *Chlamydia trachomatis* [13], and *Mycobacterium tuberculosis* [1], have been shown to induce plasma membrane defects via the secretion of protein toxins (e.g., channels that allow the entry of Ca^2+^ and other extracellular molecules). Moreover, Ca^2+^ entry has been shown to activate a classic membrane repair response [3]. Therefore, in the present study, we investigated whether *H. pylori* induces plasma membrane defects and whether this event activates a membrane repair response or additional responses relevant to the disease pathogenesis. Our study provides the first demonstration that *H. pylori* induces epithelial cell plasma membrane disruption and that a novel aspect of the repair response to this injury is increased epithelial cell proliferation.

## 2. Results and Discussion

### 2.1. *H. pylori* Infection Causes Plasma Membrane Microinjury

**Figure 1:**
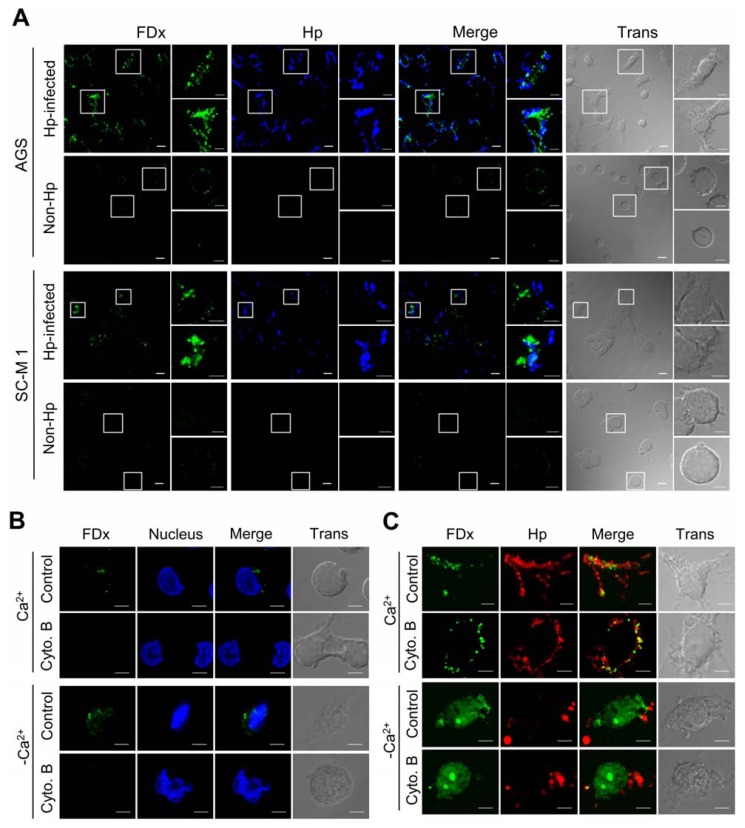
*H. pylori* infection causes plasma membrane microinjury. (**A–C**) Plasma membrane integrity was monitored in cells labeled with fluorescein isothiocyanate-dextran (FDx; green). (**A**) FDx was detected in *H. pylori*-infected AGS and SC-M1 cells, which were cultured in Ca^2+^-containing medium and infected with *H. pylori* NTUH-GC05 (Hp; blue). Scale bar, 10 μm. Scale bar in images of a single cell, 5 μm. (**B**,**C**) FDx was detected in AGS cells. (**B**) Without *H. pylori* infection and cytochalasin B (pinocytosis inhibitor) treatment, a small amount of FDx was observed to enter into non-infected cells via endocytosis in the presence or absence of Ca^2+^ (extracellular Ca^2+^ was chelated using 5 mM EGTA). The nucleus was stained with Hoechst 33258 (blue). In contrast, FDx was not observed in non-infected cells treated with cytochalasin B. Scale bar, 5 μm. (**C**) After treatment with cytochalasin B, FDx was detected in *H. pylori*-(red) infected cells, which were cultured in Ca^2+^-containing medium. In the absence of Ca^2+^, FDx was present diffusely throughout the infected cells with or without cytochalasin B treatment. Scale bar, 5 μm. Control, without cytochalasin B treatment; cyto. B, cytochalasin B; trans, transmission.

To test the hypothesis that *H. pylori* infection disrupts host plasma membrane integrity, we used membrane-impermeant fluorescein isothiocyanate-dextran (FDx) [14] as a marker to detect membrane disruption. Human gastric cancer cell lines, AGS and SC-M1, were infected with *H. pylori*; uninfected cells were used as control. FDx was observed to label the cytoplasm of cells infected with *H. pylori* NTUH-GC05 (Figure 1A, AGS Hp-infected/FDx and SC-M1 Hp-infected/FDx), but not the cytoplasm of non-infected control cells (Figure 1A, AGS and SC-M1 controls with Ca^2+^/FDx). High percentages of FDx-labeled cells were identified in infected populations (AGS, 95% ± 4.3%, *n* = 3 slides; SC-M1, 90% ± 10%, *n* = 3 slides) *vs.* control, non-infected populations (AGS, 3.7% ± 3.9%, *n* = 3 slides; SC-M, 3% ± 5.2%, *n* = 3 slides). To exclude the possibility that the presence of intracellular dextrans was due to *H. pylori* cytotoxin-induced fluid phase endocytosis or pinocytosis, we treated AGS cells with cytochalasin B, which blocks these two modes of glucose uptake [15]. In non-infected cells treated with cytochalasin B (Figure 1B, Cyto B/FDx), there was apparently less labeling with FDx than in non-infected cells not treated with cytochalasin B (Figure 1B, Control/FDx). After *H. pylori* infection, FDx could be detected as *puncta* (fluorescence hot spots) in the cytoplasm despite cytochalasin B treatment (Figure 1C). Strikingly, if Ca^2+^, which is required for a membrane repair response, was omitted from the medium in the presence or absence of cytochalasin B, the FDx labeling was of a more diffuse, global nature (Figure 1C) as against the well- defined, punctate labeling of the cytoplasm observed in the presence of Ca^2+^. These results suggest that *H. pylori* induces plasma membrane disruptions, allowing the entry of FDx into the cytoplasm of AGS and SC-M1 epithelial cells by an endocytosis-independent mechanism. However, a repair response, elicited in the presence of Ca^2+^, restricts the diffusion of FDx into the cytoplasm of gastric epithelial cells via this route.

### 2.2. Membrane Injury by *H. pylori* Infection Is Independent of VacA and CagA

**Figure 2:**
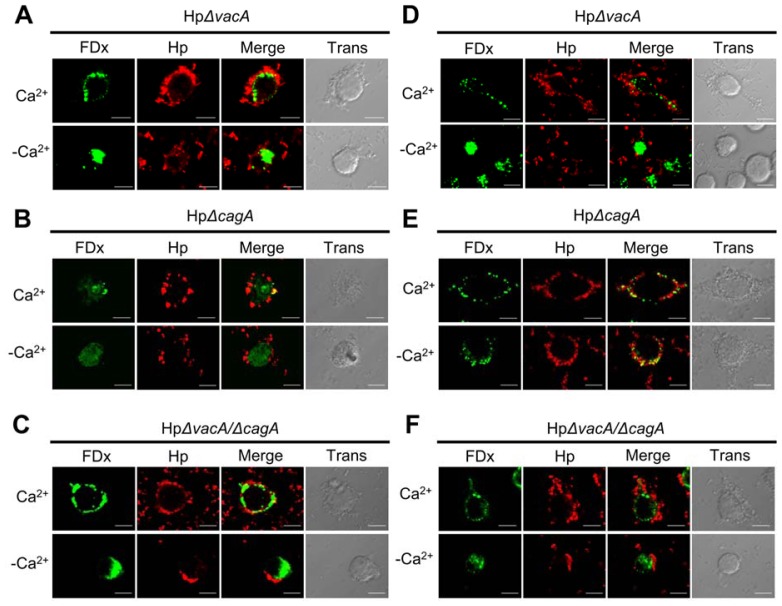
Plasma membrane injury caused by *H. pylori* is independent of VacA and CagA. The amount of FDx in the cytoplasm represents the level of membrane injury. AGS cells without cytochalasin B treatment were incubated in Ca^2+^-containing or Ca^2+^-free medium and infected with *H. pylori* (Hp; red) mutant strains (**A**) HpΔvacA, (**B**) HpΔcagA, or (**C**) HpΔvacA/ΔcagA. Cells treated with cytochalasin B are shown in (**D**–**F**). Scale bar, 10 μm. Control, without cytochalasin B treatment; cyto. B, cytochalasin B; trans: transmission.

VacA secreted by *H. pylori* causes cellular vacuolation via pinocytosis [16]. CagA induces many signaling pathways in the host cell [11]. To determine whether the entry of FDx into the cytoplasm of infected cells was mediated by VacA or CagA, the cells were infected with mutant strains (HpΔ*vacA*, HpΔ*cagA*, or HpΔ*vacA*/Δ*cagA*) (Figure 2). In the presence of Ca^2+^, FDx was present at membrane disruption sites in cells infected with mutant strains, which was consistent with the observations in cells infected with the wild-type strain. In the absence of Ca^2+^, FDx was present diffusely in the cytoplasm of the infected cells (Figure 2A–C). Cytochalasin B treatment had no effect on the patterns of FDx labeling in mutant strain-infected cells (Figure 2D–F). Thus, VacA and CagA activities did not account for the FDx entry into *H. pylori*-infected cells.

### 2.3. *H. pylori* Infection Causes Intracellular Ca^2+^ Increase and AnxA1 Translocation

**Figure 3:**
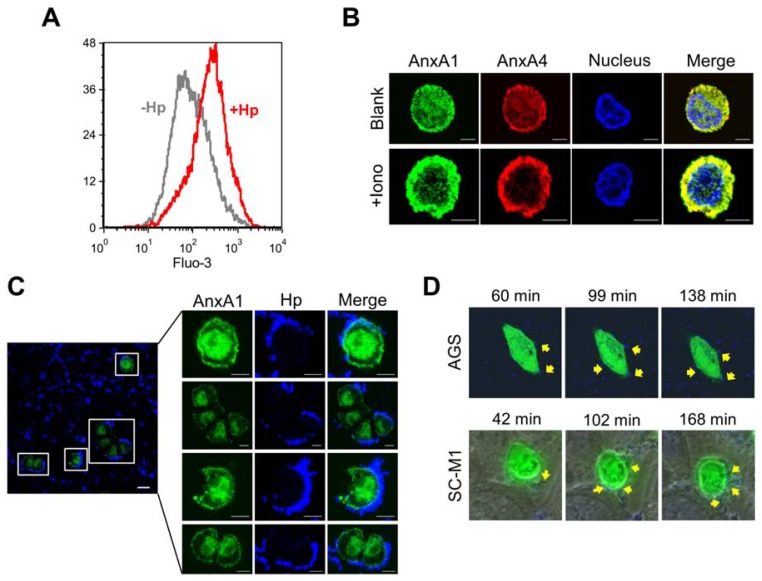
Intracellular Ca^2+^ elevation and AnxA4 localization upon *H. pylori* infection. (**A**) *H. pylori*-infected AGS cells were loaded with Fluo-3/AM to monitor intracellular Ca^2+^ levels by flow cytometry. (**B**) Immunofluorescence images showing the change in the localization of AnxA1 (green) and AnxA4 (red), indicating their more peripheral distribution in non-infected cells following treatment with a calcium ionophore (ionomycin: 5 μM). Scale bar, 5 μm. (**C**) AnxA1 (green) immunostaining localized to the plasma membrane of infected SC-M1 cells. Scale bar, 5 μm. (**D**) Dynamic localization of AnxA4 in the living cell. Real-time fluorescence images showing localization of EGFP-AnxA4 in *H. pylori*-infected AGS and SC-M1 cells (yellow arrow) stained with Hoechst 33258.

Consistent with previous reports [17], we found that the level of intracellular Ca^2+^ was increased in *H. pylori*-infected cells (Figure 3A). Annexins are a family of Ca^2+^- and phospholipid-binding proteins that have been found to exhibit a conserved core domain and play a role in membrane trafficking, vesicle aggregation, and, more recently, membrane repair [5,8,18–20]. AnxA1 was one of the first annexins to be implicated in repair [5]. In non-infected cells, AnxA1 was dispersed throughout the cell (Figure 3B, Blank/AnxA1), but it became concentrated at the cell perimeter upon treatment with a calcium ionophore (Figure 3B, +Iono/AnxA1; Supporting Information Figure S1). To determine whether *H. pylori* infection activates membrane repair, we monitored the localization of AnxA1 within infected cells. Infection with *H. pylori* induced a dramatic change in AnxA1 localization, such that the staining was observed to accumulate around the plasma membrane (Figure 3C), consistent with the translocation of AnxA1 from the cytoplasm to the plasma membrane for repair [5].

### 2.4. AnxA4 also Localizes to the Plasma Membrane after *H. pylori* Infection

AnxA4 has been reported to be overexpressed in gastric cancer patients infected with *H. pylori* [21]. To determine whether AnxA4 plays an important role in responding to *H. pylori* infection, we monitored the localization of AnxA4 in AGS and SC-M1 cells using time-lapse microscopy. Cells were transfected with enhanced green fluorescent protein (EGFP)-AnxA4 fusion protein and then infected with Hoechst-labeled *H. pylori*. In the absence of *H. pylori* infection, AnxA4 was uniformly dispersed throughout the cell, including the cytoplasm and nucleus (Figure 3B, Blank/AnxA4). Following *H. pylori* contact with the cell surface, AnxA4 gradually (over an interval of 99 to 168 minutes) accumulated at the sites of infection on the plasma membrane (Figure 3D).

### 2.5. AnxA4 Inhibits Plasma Membrane Lesions Caused by *H. pylori*

**Figure 4:**
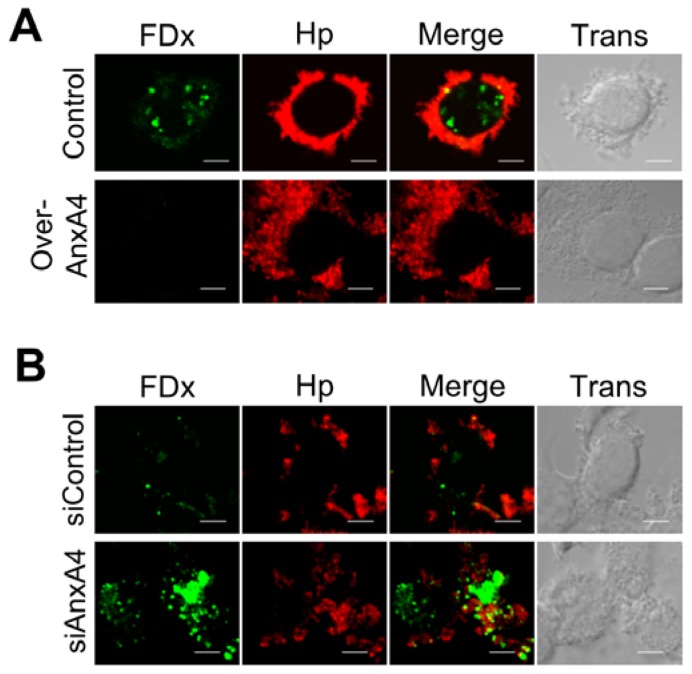
AnxA4 participates in plasma membrane repair. (**A** and **B**) Fluorescence images of FDx levels in *H. pylori* (*red*)-infected AGS cells, demonstrating the role of AnxA4 in membrane microinjury. (**A**) Overexpression of AnxA4 decreases the amount of FDx entering the cells. (**B**) In contrast, knockdown of AnxA4 increases the amount of FDx in the cell cytoplasm. Scale bar, 5 μm.

**Figure 5:**
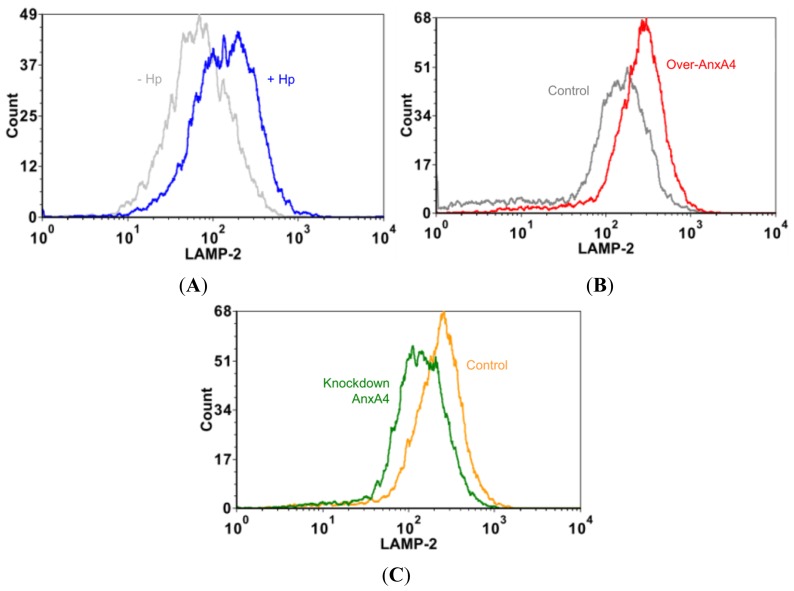
AnxA4 participates in plasma membrane repair by recruiting exocytotic membrane. (**A**) LAMP-2 fluorescence on the surface of *H. pylori*-infected AGS cells was more enhanced than on the surface of non-infected cells. (**B** and **C**) Representative flow cytometric analyses demonstrated the presence of LAMP-2 in *H. pylori*-infected cells. (**B**) AnxA4-overexpressing cells were compared with (**C**) AnxA4-silenced cells. The results indicate that AnxA4 promotes LAMP-2 expression on the surface of *H. pylori*-infected cells. AnxA4 overexpression, Over-AnxA4; Control siRNA, siControl; AnxA4 siRNA, siAnxA4.

To determine whether the observed AnxA4 translocation to the plasma membrane promoted a repair process, we overexpressed AnxA4 by transfecting cells with expression vectors or knocking down AnxA4 expression using small interfering RNAs (siRNAs). As expected, there was a 26-fold increase in FDx labeling (*n* = 15) in the cytoplasm of infected cells transfected with an empty vector over that observed in infected cells transfected with a vector encoding AnxA4 (Over-AnxA4; Figure 4A). Additionally, there was a 17-fold increase in FDx labeling (*n* = 15) in AnxA4-knockdown (siAnxA4) cells over control siRNA (siControl)-transfected cells (Figure 4B). This indicates that AnxA4 promotes membrane repair following *H. pylori* infection. Resealing of the plasma membrane is facilitated by the recruitment of intercellular vesicles derived from the endoplasmic reticulum, Golgi compartment, and lysosomes [22,23]. We found that the surface of infected cells displayed increased levels of the lysosomal marker LAMP-2 (Figure 5A). Furthermore, AnxA4 increased LAMP-2 expression on the cell surface of infected cells (Figure 5B,C). Thus, these results indicate that AnxA4 is involved in a *H. pylori*-induced repair response that displays the expected exocytotic events.

### 2.6. Membrane Disruption Promotes Cell Proliferation

**Figure 6:**
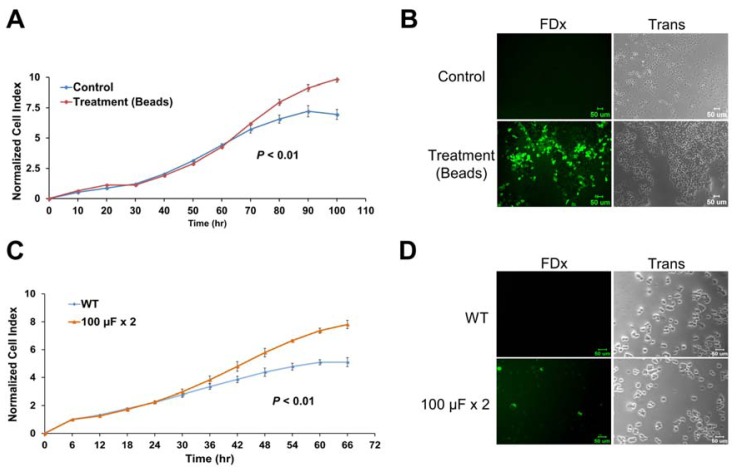
Membrane repair leads to cell proliferation. To measure cell proliferation, AGS cells were cultured in a 16-well microtiter E-plate. After incubation for 24 h, the growth rate of cells damaged with glass beads and electroporated cells were measured. It was observed that AnxA4 regulated the cell index in a time-dependent manner. (**A***–***B**) Glass beads were added to cells that were cultured for 24 h (normalized time). Then, the E-plate was rocked gently for 1 minute. Data are represented as mean ± SD; *n* = 3. (**B**) FDx can be detected in cells damaged by glass beads. (**C**–**D**) Cells were electroporated twice at 100 μF (at 5-minute intervals). Data were normalized at 6 h, which was the duration of cell adherence. Data are represented as mean ± SD; *n* = 3. (**D**) FDx can be detected in wounded cells that were stimulated by electroporation. Scale bar, 50 μm. *p* values were calculated using the two-sample Kolmogorov-Smirnov test.

Long-term chronic *H. pylori* infection could lead to increased epithelial cell proliferation, ultimately promoting the development of gastric cancer [24]. Thus, we speculate that continuing cell membrane disruptions that induce a repair response might be related to gastric carcinogenesis. To explore the relationship between membrane repair response and cell proliferation, we used glass beads and electroporation to create membrane injury and induce membrane repair responses [25,26]. FDx was detected in the cytoplasm of gastric cells that were loaded on glass beads or were electroporated, indicating membrane injury (Figure 6B,D, and Supporting Information Figure S2B). AGS cells damaged by the glass beads showed an increased growth rate compared with non-treated control cells (*p* < 0.01; Figure 6A,B). Similarly, electroporated AGS cells displayed an increased growth rate compared with non-electroporated control cells (*p* < 0.01; Figure 6C,D). The non-transformed human gastric cells Hs 738.St/Int also showed a similar response after electroporation (Supporting Information Figure S2). These results suggest that one of the repair responses to plasma membrane injury is cell proliferation.

### 2.7. Discussion

Following a bacterial infection, the activation of host defense mechanisms to reduce damage is crucial for host survival. Recent reports have shown that host cells are actively involved in membrane repair responses to bacterial infections. Host cells have been reported to induce plasma membrane repair response to membrane permeabilization caused by two human gastrointestinal pathogens, *Salmonella enterica* serovar Typhimurium and *Yersinia pseudotuberculosis* [3]. To avoid host immune responses, *Mycobacterium tuberculosis*, a virulent lung pathogen, impairs plasma membrane repair [1].

*H. pylori* is a human gastric pathogen and a risk factor for gastric malignancy. However, the link between *H. pylori* and plasma membrane repair response is currently unknown. Here, cytochalasin B-resistant staining of the cytoplasm with a membrane-impermeant probe, FDx, develops sites of *H. pylori* interaction with the plasma membrane of host gastric cells. This staining was observed after infection with both wild-type and virulence factor-deficient *H. pylori* strains. It is worth noting that the FDx-labeling pattern produced as a result of *H. pylori*-induced membrane disruptions was punctate and localized to the site of bacterium/cell interaction (Figures 1 and 2), whereas the FDx-labeling pattern obtained through larger plasma membrane disruptions, such as those produced by glass beads (Figure 6B), was diffuse and more global. Similar phenomena were observed in damaged BS-C-1 cells and sea urchin eggs [27,28].

We further propose that *H. pylori* induces Ca^2+^ entry and consequent membrane fusion events. These results mimic the findings from an earlier report: when Ca^2+^ was injected into the cell cytoplasm via a microneedle, the Ca^2+^-containing solution was sequestered behind a newly formed membrane barrier as it left the microneedle [29]. This barrier was presumed to be formed via homotypic fusion events involving one or more cytoplasmic organelles.

Some virulence factors used by *H. pylori* have been reported that they can cause the disruption of cell junction structures, e.g., CagA [12,30] and urease [31]. Lai *et al*. and Amieva *et al.* have shown that the disruption of apical-junctions in primary gastric epithelial cells can be caused by CagA [12,30]. Moreover, the disruption of the tight junctions in cells can be caused by ammonium which is produced by *H. pylori* urease [31]. A recent study has shown that the bacterial toxin streptolysin O (SLO) can induce endocytosis to remove the lesions of the plasma membrane [32]; however, *H. pylori* does not possess SLO. Although in the present study we demonstrated that neither VacA nor CagA is involved in membrane disruption, the mechanism by which sequestration is mediated in *H. pylori*-infected cells remains to be elucidated.

AnxA1 participates in the plasma membrane repair response and exhibits a Ca^2+^ sensitivity similar to that of AnxA4 for translocation to the plasma membrane [5,33]. In the present study, we showed for the first time that AnxA4 is involved in plasma membrane repair; it localizes to the membrane disruption sites, and repair is apparently enhanced by AnxA4 overexpression and inhibited by gene silencing. We also found that AnxA4 overexpression significantly increases cell growth rate, whereas AnxA4 knockdown significantly decreases the growth rate (Supporting Information Figure S3). In a previous study, we showed that AnxA4 expression is increased in *H. pylori*-associated tumors [21]. Moreover, we have shown that disruption of the plasma membrane induced by glass beads or electroporation can activate epithelial cell proliferation.

These results suggest that long-term *H. pylori*-induced plasma membrane repair could have oncogenic potential. In previous studies, AnxA4 has been reported that is associated with anti-apoptosis and a biomarker of colorectal cancer for tumor diagnosis [34,35]. It also has been observed to be overexpressed in other cancers, including prostate cancer [36], renal carcinoma [37], pancreatic adenocarcinoma [38] and clear cell carcinoma of ovary [39,40]. In this study, the altered location and expression of AnxA4 could be considered as a marker for *H. pylori*-induced plasma membrane repair response. We suggest that targeting of AnxA4 might be a new therapeutic strategy for *H. pylori*-induced carcinogenesis. This study could provide a new direction in cancer-drug development.

## 3. Experimental Section

### 3.1. Bacterial Strains and Culture Conditions

The *H. pylori* (NTUH-GC05) strain (*vacA*+/*cagA*+) from the stomach of a male gastric cancer patient at the National Taiwan University Hospital was obtained in 1991. The *H. pylori* strain NTUH-GC05 and GC05Δ*cagA* were collected from the National Taiwan University Hospital (NTUH), as described elsewhere [12,41]. *H. pylori* was grown on Columbia blood agar base (BD Difco) containing 5% sheep blood and incubated for 2–3 days in microaerophilic conditions (5% O_2_, 10% CO_2_, 85% N_2_) at 37 °C.

### 3.2. Cell Lines and Culture Conditions

Human stomach adenocarcinoma AGS cells (CRL-1739, ATCC) and SC-M1 cells (cultured from a poorly differentiated adenocarcinoma that showed no metastasis to lymph nodes or adjacent organs) [42] were grown in 90% RPMI 1640 medium (Biological Industries) supplemented with 1% penicillin/streptomycin and 10% fetal bovine serum (Biological Industries). Hs 738.St/Int fibroblast cells (CRL-7869, ATCC) are non-transformed, human fetal gastric/intestinal cells and were grown in 90% Dulbecco’s modified Eagle’s medium (Gibco-Invitrogen) with 10% fetal bovine serum. Cells were cultured at 37°C in a controlled humidified atmosphere in an incubator containing 5% CO_2_.

### 3.3. Construction of Insertion Mutants of *H. pylori*

To generate a *vacA* mutant and a *vacA*/*cagA* double mutant, the *vacA* gene was amplified using PCR with the primer pair *vacA*-F and *vacA*-R (Supporting Information Table S1), and the resulting fragments were cloned into a pGEM-T easy vector (Promega, Madison, WI, USA). The cloned *vacA* gene was then digested with EcoRV, dephosphorylated, and finally ligated to the kanamycin resistance gene. The resulting plasmid was introduced by natural transformation into *H. pylor*i GC05 and GC05Δ*cagA* [41]. The *vacA* insertion mutants were selected using 10 μg/mL kanamycin and verified by PCR using the primer pair *vacA*-F2 and *vacA*-R2.

### 3.4. Plasmids and Transfections

Full-length *anxa4* was amplified by PCR using the primer pair *anxa4*-F and *anxa4*-R, and the amplification product was inserted into the HindIII/EcoRI sites of pcDNA 3.1(+) (Invitrogen, Burlington, ON, USA). For immunofluorescence analysis, *anxa4* was amplified by PCR using the primer pair *anxa4*-F2 and *anxa4*-R2, and the amplification product was inserted into the HindIII/pst I sites of pEGFP-C1 (BD Clontech, Palo Alto, CA, USA). AnxA4-specific siRNA and negative control Stealth siRNA (Stealth™ RNAi) were purchased from Invitrogen. Cells were cultured in six-well plates or on coated cover slips for 24 h. Cells were then transiently transfected with pcDNA 3.1(+)/pEGFP-C1/AnxA4 (8 μg for a six-well plate; 0.4 μg/mL for a 96-well E-plate) or AnxA4 siRNA (100 pmoles for a six-well plate; 10 pmoles for a 96-well E-plate) using Lipofectamine 2000 (Invitrogen, Burlington, IA, USA) according to the manufacturer’s instructions. The efficiency of expression vector and siRNA transfection was determined by immunoblotting. After transfection for 48 h, the differential expression of proteins and genes was detected.

### 3.5. Antibodies

The mouse monoclonal antibodies used in this study were as follows: AnxA1 (sc-12740) from Santa Cruz Biotechnology; LAMP-2 (ab25631) from Abcam; IgG_1_ isotype (555746) from BD Biosciences; and α-tubulin (T5168) from Sigma. The rabbit polyclonal antibodies were as follows: *H. pylori* (AHP602H) from AbD Serotec. The goat polyclonal antibody AnxA4 (sc-1930) was from Santa Cruz Biotechnology.

### 3.6. Live Cell Imaging

To determine AnxA4 localization after *H. pylori* infection, AGS or SC-M1 cells were plated on Lab-Tek™ chamber slide™ system (Nunc, Roskilde, Denmark). After 24-h incubation, cells were transfected with pEGFP-C1/AnxA4 for 48 h prior to infection. *H. pylori* was resuspended in serum-free culture medium and stained with Hoechst 33258 (Sigma) for 1 h. The stained cells were centrifuged, washed twice, and then resuspended in fresh serum-free culture medium. Cultured cells were replaced with fresh serum-free culture medium (1 mL per well) and infected with *H. pylori* at a multiplicity of infection (MOI) of 150. Fluorescence images of living cells were captured by fluorescence microscopy (SC-M1: Zeiss Axiovert 200M (Zeiss); AGS: Nikon A1 confocal microscope (Nikon)). Images were processed using MetaMorph software (Version 7.7; Molecular Devices: Sunnyvale, CA, USA, 2010).

### 3.7. Confocal Microscopy

Cells (8 × 10^5^ cells/well) were cultured on glass cover slips coated with poly-l-lysine in six-well plates. To observe plasma membrane-disruption sites, cells were treated with cytochalasin B (10 μg/mL) for 1 h prior to infection and then supplemented with fluorescein isothiocyanate-dextran (FDx) (5 mg/mL; 10 kDa) for 30 minutes after infecting the cells with *H. pylori* for 2.5 h. To examine the effect of the absence of Ca^2+^ on *H. pylori*-induced plasma membrane disruptions, cells and *H. pylori* were co-cultured in Ca^2+^-free medium, which was prepared by adding the Ca^2+^ chelator EGTA (5 mM) to remove extracellular calcium from the medium. To determine the cellular location of AnxA1 and AnxA4 in the presence of high intracellular [Ca^2+^]i, the Ca^2+^ ionophore, ionomycin (5 μM), was added to the cells. After infection (MOI = 150) for 3 h, cells were fixed with 4% paraformaldehyde for 15 minutes, permeabilized with 1% Triton-X-100 in PBS (to observe the location of AnxA1 and AnxA4) for 30 minutes, and blocked with 0.1% BSA/PBS overnight at 4 °C. Cells were incubated with mouse anti-AnxA1 antibody (1:100), goat anti-AnxA4 antibody (1:100), or rabbit anti-*H. pylori* antibody (100 μL) for 1 h at RT. After washing, FITC-labeled anti-mouse (Sigma), TRITC-labeled anti-goat (Sigma), or Cy5-labeled anti-rabbit (Millipore) were used as secondary antibodies at a 1:100 dilution and applied for 1 h at RT. The cover slips were then washed and mounted onto slides. Immunostaining of the cells was observed using confocal microscopy with a Plan-Apochromat 63x/1.40 oil M27 objective (Zeiss LSM 510). Images were analyzed using MetaMorph (Molecular Devices) software.

### 3.8. Flow Cytometry

To measure intracellular [Ca^2+^]i, cells were infected (MOI = 150) for 3 h, and both infected and non-infected cells were washed in Hanks’ balanced salt solution (HBSS) buffer (137 mM NaCl, 5.33 mM KCl, 4.2 mM NaHCO_3_, 0.44 mM KH_2_PO_4_, 0.34 mM Na_2_HPO_4_, 5.56 mM glucose; pH 7.3) and then loaded with 4 μM of the Ca ^2+^ indicator, Fluo-3-AM/pluronic acid F-127, for 1 h at 37 °C. Subsequently, cells were harvested using trypsin and then resuspended in HEPES buffer (137 mM NaCl, 5 mM KCl, 1 mM Na_2_HPO_4_, 5 mM glucose, 1 mM CaCl_2_, 0.5 mM MgCl_2_, 1 mg/mL BSA, and 10 mM HEPES, pH 7.4). Cells cultured in the presence of ionomycin (5 μM) were used as a positive control for increased Ca^2+^ concentration in the cytoplasm. To determine the cell surface expression of LAMP-2, cells were resuspended in PBS and then fixed with 2% paraformaldehyde, centrifuged, washed in PBS, and blocked with 2% BSA/PBS for 15 minutes. The mouse anti-LAMP-2 antibody and mouse IgG_1_ isotype control were used at a dilution of 1:200 for 30 minutes at RT, following which the cells were washed and stained with anti-FITC secondary antibody for 30 minutes at RT. Cells were finally washed and fixed with 4% paraformaldehyde. Fluorescence intensity was determined using the FACSCalibur System (BD Biosciences, San Diego, CA, USA), and the data were acquired by analyzing at least 10,000 cells from each sample.

### 3.9. Cell Proliferation Assay

AGS cells (1 × 10^4^ cells/well for glass bead stimulation; 1.5 × 10^4^ cells/well for electroporation) and Hs 738.St/Int cells (1 × 10^3^ cells/well) were loaded in each well of a 16-well microtiter E-plate. Each well contained microelectronic sensor arrays at the base to detect the cell index (CI). For transfection experiments, after incubation for 24 h, AGS cells were transfected with expression vectors or siRNAs for 6 h and monitored for a total of 84 h. AGS cells were resuspended in RPMI medium, and Hs 738.St/Int cells were resuspended in DMEM medium containing 10% FBS. The cells (4 × 10^5^ cells in a 400-μL cell suspension) were placed in 0.2-cm cuvettes (Bio-Rad, Hercules, CA, USA) and electroporated twice at 200 V and 100 μF at 5-minute intervals. After electroporation, cells were incubated with 5 mg/mL of FDx for 5 minutes to allow for analysis of cell membrane damage by fluorescence microscopy. Glass beads (Sigma, St. Louis, MO, USA) were used to create membrane injury as previously described [26], with slight modifications. Briefly, glass beads (bead size, 450–600 μm; bead weight, 0.03 g/well in E-plate) were carefully placed onto a plate and gently rocked for 1 minute. The E-plate was placed in the Real-Time Cell Analyzer (RTCA) system and incubated in an incubator containing 5% CO_2_ at 37 °C. The level of cell proliferation was represented as CI, which was based on the electrical impedance measured using the xCELLigence system (Roche, Penzberg, Germany).

### 3.10. Statistical Analysis

Data were expressed as mean ± standard deviation (SD). Difference between independent groups was analyzed using a two-tailed Student’s *t* test. Data obtained from the cell proliferation assay were analyzed using the two-sample Kolmogorov-Smirnov test. A *p* value of 0.05 indicated statistical significance.

## 4. Conclusions

Our results show that *H. pylori* infection can cause plasma membrane disruption that is independent of VacA and CagA. AnxA1 and AnxA4 are involved in an epithelial cell membrane repair response induced by *H. pylori*-generated plasma membrane disruptions. AnxA4 can increase plasma membrane repair and be an indicator of *H. pylori*-induced plasma membrane disruption. Plasma membrane disruption and AnxA4 can promote cell proliferation. This study indicates that pathogen-induced plasma membrane repair response could be a new risk factor for carcinogenesis. The insight could provide a different direction for the future research of *H. pylori*-related diseases. We suggest that *H. pylori*-generated plasma membrane disruptions might serve as pathogenic events in *H. pylori*-induced carcinogenesis, and AnxA4 could be a new drug target for cancer treatment.

## Supplementary Materials
