# Supplementary material for: Helicobacter pylori Disrupts Host Cell Membranes, Initiating a Repair Response and Cell Proliferation
Source: Int J Mol Sci. 2012 Aug 15;13(8):10176–92. doi: 10.3390/ijms130810176 (PMC3431852; doi:10.3390/ijms130810176)
Supplement: Supplementary file 1 [file ijms-13-10176-s001.pdf]

Figure S1. Cells were treated with ionomycin (a calcium ionophore). The increase in cellular Ca^{2+} levels was measured by flow cytometry.

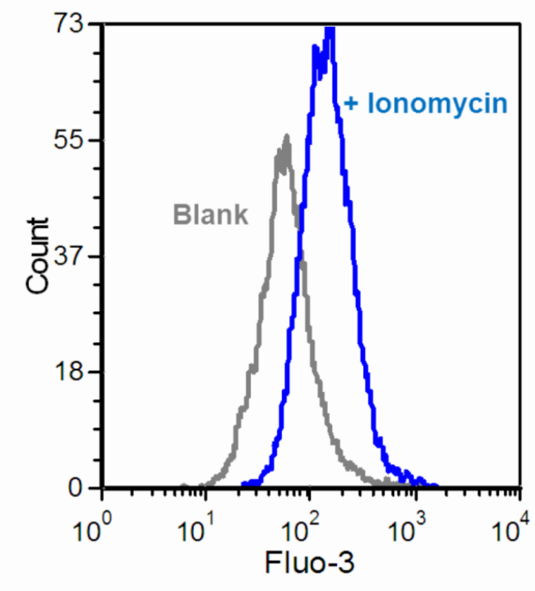

Figure S2. Membrane repair leads to proliferation of Hs 738.St/Int cells. **(A)** Hs 738.St/Int cells were electroporated twice at 100 μF (at 5-minute intervals). Cells (1×10^3 cells/well) were plated in a 16-well microtiter E-plate. Data were normalized at 10 h, which was the duration of cell adherence. $P < 0.05$ vs. control treatment values. **(B)** FDx (green fluorescence) can be detected in damaged cells, but not in wild-type (WT) which were non-electroporated cells, suggesting membrane injury due to electroporation. The damaged cells participate in a membrane-repair response that leads to cell proliferation (bottom right panel). Scale bar, 50 μm. Trans, transmission.

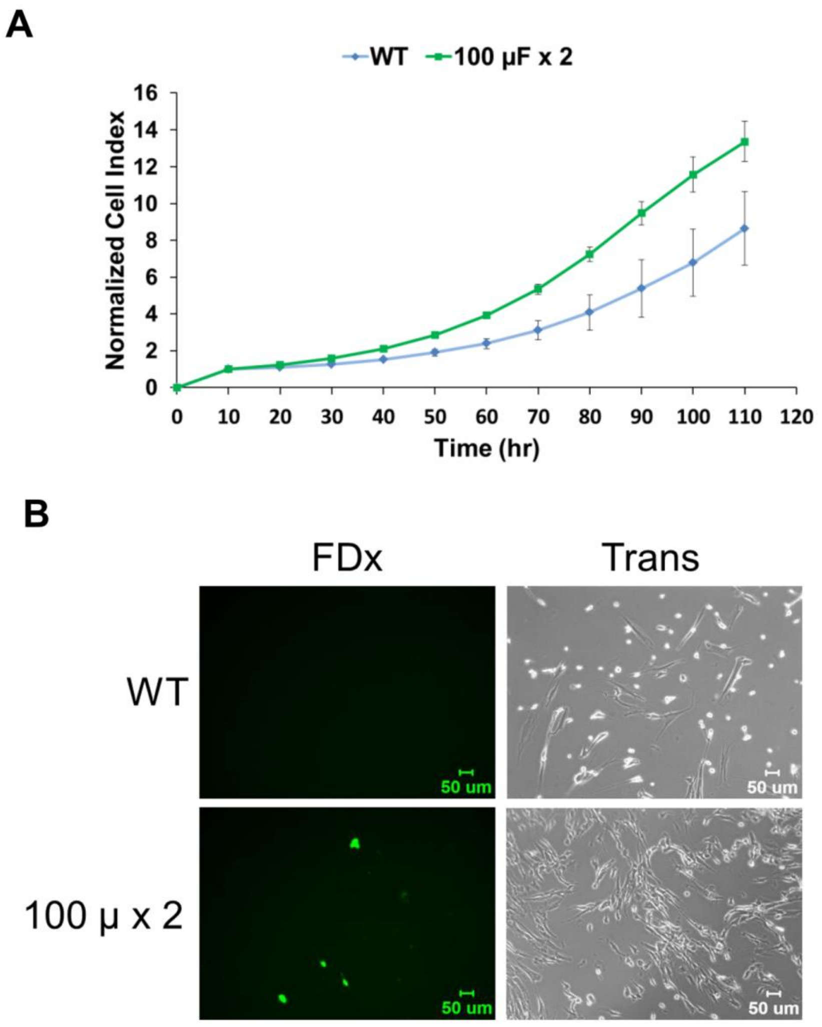

Figure S3. AnxA4 promotes cell proliferation. After incubation for 24 h, the cell growth rate of (A) cells overexpressing AnxA4, and (B) cells containing AnxA4-specific siRNA were measured. It was observed that AnxA4 regulated the cell index in a time-dependent manner. (A and B) Data were normalized from measurements taken at 24 h, which was when transfection was initiated. The detection time from three independent experiments is represented as mean ± SD, n=3. *P* values were calculated using the two-sample Kolmogorov-Smirnov test.

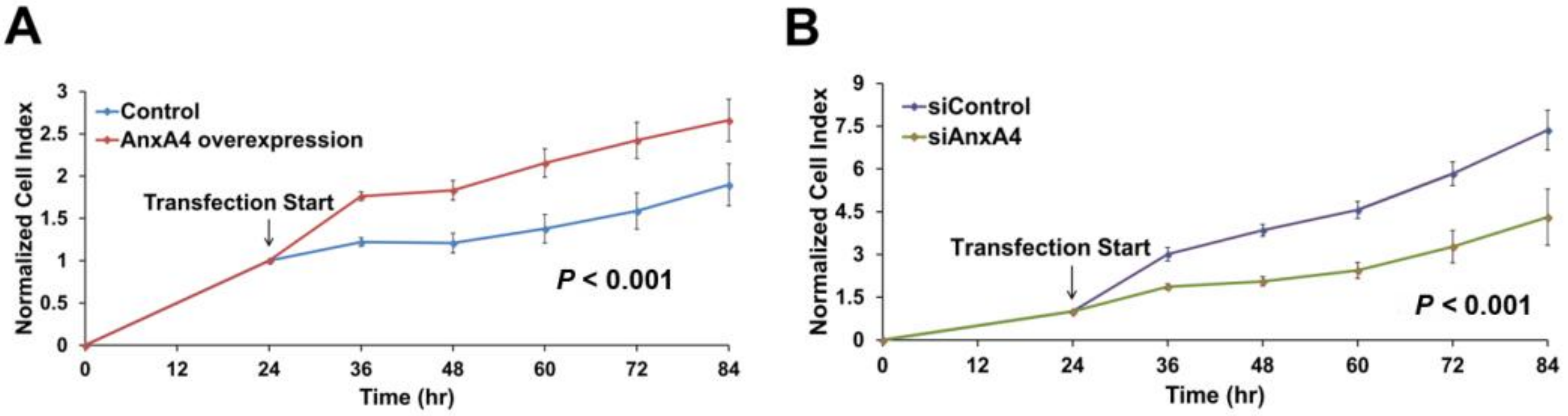

Table S1. List of primer sequences used for construction.

Primer	Sequence (5’→3’)	Purpose
anxa4-F	atataagcttgccaccatggccatggcaaccaaa	Constructing pcDNA 3.1(+)/AnxA4
anxa4-R	gcgcgggaattcttaatcatctcctccaca	Constructing pcDNA 3.1(+)/AnxA4
anxa4-F2	atataagcttgccaccatggccatggcaaccaaa	Constructing pEGFP-C1/AnxA4
anxa4-R2	agcgcgcctgcagttaatcatctcctccaca	Constructing pEGFP-C1/AnxA4
vacA-F	gagtgaataatcaagtgggtgg	Constructing *vacA* mutant
vacA-R	tcatcgcattactcaagctcaa	Constructing *vacA* mutant
vacA-F2	ggcacgattaaagtgggagg	Checking *vacA* mutant
vacA-R2	gttagcccaaacattggtagg	Checking *vacA* mutant
